# GSK3β Controls mTOR and Prosurvival Signaling in Neurons

**DOI:** 10.1007/s12035-017-0823-9

**Published:** 2017-11-15

**Authors:** Malgorzata Urbanska, Agata Gozdz, Matylda Macias, Iwona A. Cymerman, Ewa Liszewska, Ilona Kondratiuk, Herman Devijver, Benoit Lechat, Fred Van Leuven, Jacek Jaworski

**Affiliations:** 1 Laboratory of Molecular and Cellular Neurobiology, International Institute of Molecular and Cell Biology, Ks. Trojdena St. 4, 02-109 Warsaw, Poland; 2 Department of Neurology and Epileptology, Children’s Memorial Health Institute, 04-730 Warsaw, Poland; 3 Laboratory of Neurobiology, The Nencki Institute of Experimental Biology, 02-093 Warsaw, Poland; 4 Department of Human Genetics, Experimental Genetics Group – LEGTEGG, KU Leuven, 3000 Leuven, Belgium

**Keywords:** Glycogen synthase kinases-3, Mammalian target of rapamycin, Neurons, Neuronal survival, Excitotoxicity

## Abstract

Glycogen synthase kinases-3β (GSK3β) is a key regulator of cell homeostasis. In neurons, GSK3β contributes to control of neuronal transmission and plasticity. Despite extensive studies in non-neuronal cells, crosstalk between GSK3β and other signaling pathways remains not well defined in neurons. In the present study, we report that GSK3β positively affected the activity of effectors of mammalian target of rapamycin complex 1 (mTORC1) and complex 2 (mTORC2), in mature neurons in vitro and in vivo. GSK3β also promoted prosurvival signaling and attenuated kainic acid-induced apoptosis. Our study identified GSK3β as a positive regulator of prosurvival signaling, including the mTOR pathway, and indicates the possible neuroprotective role of GSK3β in models of pharmacologically induced excitotoxicity.

**Electronic supplementary material:**

The online version of this article (10.1007/s12035-017-0823-9) contains supplementary material, which is available to authorized users.

## Introduction

The glycogen synthase kinase-3 (GSK3) family of protein kinases is crucially important for cell metabolism and homeostasis [1, 2]. In mammals, two genes encode GSK3 proteins: *GSK3α* and *GSK3β*. GSK3α and GSK3β are structurally similar with partially overlapping substrate specificity and cellular functions, although these remain ill defined [1, 2]. Among the downstream effectors of GSK3α and GSK3β are mammalian target of rapamycin (mTOR) complexes 1 and 2 (mTORC1 and mTORC2), key regulators of cell metabolism and the cytoskeleton [3]. Several molecular pathways that underlie the GSK3 isozyme-dependent inhibition of mTOR have been described [4, 5] but in some cases, GSK3α and GSK3β were shown to positively regulate the mTORC1 pathway, e.g. MCF7 and neuroblastoma SH-SY5Y [6–8]. Nevertheless, unknown to date is whether such positive regulation occurs only in selected cell lines or may also exist in highly differentiated cells, such as neurons.

GSK3β as well as mTOR are implicated in neuronal survival. For example, in neurons cultured in vitro inhibition or knockdown of GSK3β typically protects against proapoptotic stimuli (e.g., trophic factors withdrawal, neuronal activity deprivation) [9, 10]. Consequently, overexpression of GSK3β in mature forebrain neurons in vivo resulted in increased apoptosis of neurons [11, 12]. But role of GSK3β in neuronal survival is not a clear-cut. For example, in vivo overexpression of kinase-dead GSK3β induced neuronal apoptosis similarly to overexpression of wild type GSK3β [13]. What is more, transgenic overexpression of GSK3β S9A mutant, considered to act as constitutively active kinase, because it cannot be inhibited by upstream kinases (e.g., Akt), did not cause any cell death [14, 15]. Furthermore, substitution of wild type *GSK3* to *GSK3S9A* by knock-in strategy did not induce neuronal apoptosis [16]. Also, mTOR role in neuronal survival is not fully elucidated. Both, insufficient and excessive mTORC1 activation were reported proapoptotic either for neural progenitors or fully developed neurons, depending on experimental paradigm [17, 18].

In the present study, we investigated whether GSK3 isozymes positively affect the mTOR signaling network in neurons. We report that under basal conditions GSK3β is needed for proper mTORC1 and mTORC2 signaling in mature neurons in vitro. What is more we show that in vivo transgenic expression of constitutively active GSK3β (GSK3β S9A) enhanced mTORC1 and mTORC2 activation in response to increased neuronal transmission. We also present evidence that transgenic expression of GSK3β S9A increased the activity of prosurvival signaling pathways and partly protected neurons from KA-induced neurotoxicity.

## Methods

### Antibodies, DNA Constructs, and Reagents for Pharmacological Treatments

The following mammalian expression plasmids were described previously: pSUPER^GFP^ and pSUPER^GFP^ shGSK3β#12-#15 [19]. The antibodies that were used for this study are listed in Table 1. Alexa Fluor 594-conjugated secondary antibody (donkey anti-rabbit; catalog no. A-21207) was purchased from Invitrogen (Carlsbad, CA, USA). Horseradish peroxidase-conjugated anti-rabbit and anti-mouse secondary antibodies (goat anti-rabbit IgG, catalog no. 111-035-003; goat anti-mouse IgG, catalog no. 115-035-003) and IRDye-conjugated secondary antibodies (IRDye 680LT donkey anti-rabbit antibody, catalog no. 926-68023; IRDye 800CW donkey anti-mouse antibody, catalog no. 926-32212) were obtained from Jackson ImmunoResearch (West Grove, PA, USA) and LI-COR Biosciences (Lincoln, NE, USA), respectively. Secondary antibodies were used in dilutions that were recommended by the manufacturers. The following reagents were used for the pharmacological treatments: DMSO (Sigma-Aldrich, St. Louis, MO, USA), Chiron 98014 (Ch98, Axon Medchem, Groningen, The Netherlands), BIO (Tocris Bioscience, Ellisville, MO, USA), kainic acid (KA, Tocris Bioscience), brain-derived neurotrophic factor (BDNF, Sigma-Aldrich), insulin (Sigma-Aldrich), and pentylenetetrazole (PTZ, Sigma-Aldrich).

**Table 1:** Primary antibodies

Antigen	Manufacturer	Catalog no.	Host	Dilution (application)
P-S6 ribosomal protein (S235/S236)	Cell Signaling Technology (Danvers, MA, USA)	4858	Rabbit	1:1000 (WB) 1:150 (IF)
P-S6 ribosomal protein (S240/S244)	Cell Signaling Technology	5364	Rabbit	1:1000 (WB)
S6 ribosomal protein	Cell Signaling Technology	2317	Mouse	1:1000 (WB)
P-4E-BP1 (T37/T46)	Cell Signaling Technology	2855	Rabbit	1:250 (WB)
4E-BP1	Cell Signaling Technology	9452	Rabbit	1:1000 (WB)
P-Akt (S473)	Cell Signaling Technology	4060	Rabbit	1:1000 (WB)
P-Akt (T308)	Cell Signaling Technology	13,038	Rabbit	1:2000 (WB)
Akt	Cell Signaling Technology	2920	Mouse	1:1000 (WB)
P-Glycogen synthase (S641)	Cell Signaling Technology	3891	Rabbit	1:1000 (WB)
Glycogen synthase	Cell Signaling Technology	3893	Rabbit	1:1000 (WB)
P-NDRG1 (T346)	Cell Signaling Technology	5482	Rabbit	1:1000 (WB)
NDRG1	Cell Signaling Technology	5196	Rabbit	1:1000 (WB)
P-p44/42 MAPK (Erk1/2) (T202/Y204)	Cell Signaling Technology	9101	Rabbit	1:2000 (WB)
p44/42 MAPK (Erk1/2)	Cell Signaling Technology	9102	Rabbit	1:1000 (WB)
P-FAK (Y925)	Cell Signaling Technology	3284	Rabbit	1:1000 (WB)
P-FAK (Y576/Y577)	Cell Signaling Technology	3281	Rabbit	1:1000 (WB)
FAK	Cell Signaling Technology	3285	Rabbit	1:1000 (WB)
P-p38 MAPK (T180/Y182)	Cell Signaling Technology	4511	Rabbit	1:1000 (WB)
p38 MAPK	Cell Signaling Technology	9212	Rabbit	1:1000 (WB)
α-tubulin	Sigma-Aldrich (Saint Louis, MO, USA)	T5168	Mouse	1:5000 (WB)

*WB* Western blot, *IF* immunofluorescence

### Animals

Pregnant wildtype FVB mice were obtained from the Mossakowski Medical Research Centre, Polish Academy of Sciences. GSK3β S9A transgenic mice and GSK3β neuron-specific knockout mice (GSK3β[n−/−]) were described previously [14, 15, 20]. GSK3β S9A mice carry a constitutively active form of GSK3β, with a mutation of Ser9 to Ala, with the transgene under control of the mouse *Thy-1* gene promoter. Heterozygous GSK3β S9A mice were maintained on an FVB/N genetic background and compared with wildtype littermates as controls. GSK3β(n−/−) mice were obtained by crossing mice with floxed GSK3β alleles (GSK3β^fl/fl^) with Thy1-Cre recombinase transgenic mice. GSK3β(n−/−) mice were maintained on a mixed FVB-C57BL/6 genetic background and compared to GSK3β^fl/fl^ littermates as controls. In experiments described herein the following numbers of each genotype animals were used: the Western blot analysis of phospho-proteins levels under basal conditions (wild type [WT] - 6, GSK3β S9A - 5), the Western blot analysis phospho-proteins levels in response to PTZ (WT + NaCl - 5, WT + PTZ - 6, GSK3β S9A + NaCl - 6, GSK3β S9A + PTZ - 6, GSK3β[n−/−] + NaCl - 3, GSK3β[n−/−] + PTZ - 3, GSK3β^fl/fl^ + NaCl - 3, GSK3β^fl/fl^ + PTZ - 4), TUNEL staining analysis of KA-treated organotypic slices (wild type [WT] - 7, GSK3β S9A - 10).

### Primary Cortical and Hippocampal Neuron Culture Preparation, Transfection, and Drug Treatment

Hippocampal and cortical neurons for in vitro cultures were obtained from wildtype embryonic mouse brains as described previously [19]. To obtain neurons for tissue culture, pregnant mouse females and embryonic day 16 embryos (both sexes) were sacrificed according to a protocol that was approved by the 1st Ethical Committee in Warsaw, Poland (decision no. 188/2011), which is in compliance with the European Community Council Directive (86/609/EEC). To inhibit GSK3 on day in vitro (DIV) 14, cortical neurons were treated with Ch98 (1 and 5 μM) or BIO (1 and 5 μM) dissolved in DMSO. Drugs were added to the growth medium 2 h before lysis or treatment with BDNF (100 ng/ml) or insulin (400 nM). For BDNF and insulin treatment, the cells were first starved of trophic factors for 24 h in neuron culture media with a reduced amount of B27 (0.2%). To silence GSK3β expression, neurons were transfected with Lipofectamine2000 (Invitrogen) on DIV14 using pSUPER^GFP^ shRNA constructs as described previously [19]. The efficiency of transfection typically reached 1–2%.

### Western Blot Analysis

Western blot (WB) was performed as described previously [21–23]. For quantitative analysis, we used WB signal detection with Infrared Odyssey Imaging System (LI-COR Biosciences). Towards this end membranes with transferred proteins, after incubation with primary antibodies, were washed 3 times with TBS-T (10 mM Tris, pH 8.0, 150 mM NaCl, 0.1% Tween-20) and incubated with appropriate IRDye680 and IRDye800 secondary antibodies in TBS-T with 5% nonfat milk for 1 h. Next, blots were washed with TBS-T and deionized water. Dried membranes were used for fluorescence signal acquisition with use of Infrared Odyssey Imaging System and the results were quantified using Image Studio™ Lite (LI-COR Biosystems). Signal intensity from phosphorylated and total protein was firstly normalized to respective tubulin levels and then the phosphorylated/total protein ratio was calculated.

### Immunofluorescence, Image Acquisition, and Analysis

For the immunofluorescent staining of P-rpS6, neurons were fixed with 4% paraformaldehyde that contained 4% sucrose in phosphate-buffered saline for 10 min at room temperature. Staining was then performed according to the manufacturer’s protocol (Cell Signaling Technology). For fluorescence intensity analysis, confocal cell images were obtained at a 1024 × 1024 pixel resolution using a Zeiss LSM710 NLO microscope with a 20× objective. Each image was a z-series of images, each averaged twice per line. The obtained stack was “flattened” into a single image using a maximum intensity projection. The confocal settings were kept the same for all scans when the fluorescence intensities of immunostaining in neuronal cell somas were compared. Quantification was performed as described previously [24].

### Organotypic Mouse Hippocampal Slices

Hippocampal slice cultures were prepared from 5-day-old mice according to a previously described method [25]. The maintenance medium was changed every 3–4 days. For the pharmacological treatments, the slices were treated with either vehicle (DMSO) or 10 μM KA on DIV10-14. The slices were collected 24 h after treatment.

### Pentylenetetrazole Treatment

The mice (3.5–4 months old) were habituated to handling and intraperitoneal (i.p.) injections of 0.9% NaCl twice per day for 7 days. On day 8, the mice were divided into groups that received either PTZ (50 or 30 mg/kg, i.p.) or vehicle (0.9% NaCl, i.p.). The mice were sacrificed by cervical dislocation 10 or 15 min after the onset of PTZ-induced seizures or after the saline injection. The researchers were authorized and supervised by the University Animal Welfare Commission (Ethische Commissie Dierenwelzijn, KULeuven, Leuven, Belgium).

### Terminal Deoxynucleotidyl Transferase dUTP Nick End Labeling Assay (TUNEL)

The TUNEL assay was performed using the In Situ Cell Death Detection Kit, TMR red (Roche Applied Science, Mannheim, Germany), as described previously [25]. TUNEL staining was performed on two independent slice cultures. Slices from each animal were exposed to all types of treatment. At least three slices were analyzed per animal per condition. A Nikon Eclipse 80i microscope equipped with a monochromatic charge-coupled device Evolution VF camera (Media Cybernetics, Rockville, MD, USA) and Image-Pro Plus 5.0 software (Media Cybernetics Inc.) were used for image acquisition and whole-slice reconstruction. Then Imaris 8.2 software (Bitplane, Zurich, Switzerland) was used for quantification of TUNEL positive nuclei. First red channel with TUNEL positive nuclei was selected and used for creating the mask covering all stained nuclei per slice, which were next automatically counted. The mean number of TUNEL positive nuclei per slice was calculated. The slices were additionally counterstained with anti-P-S235/S236 rpS6 antibody to verify the effectiveness of KA-induced stimulation.

### Statistical Analysis

For immunofluorescence analysis, the data were obtained from three independent batches of neurons. The exact numbers of cells are provided in the respective figure legends. For quantification of the Western blot analysis, each cell culture or animal represents one biological repetition and number of cell cultures/animals used for experiments are provided in respective figure legends. For the TUNEL analysis, each animal (at least three slices per condition) corresponds to *n =* 1. The statistical analyses were performed using Prism software (GraphPad, San Diego, CA, USA) and the unpaired *t* test, one sample *t* test, or two-way ANOVA with Bonferroni correction. The exact test used and number of analyzed samples are indicated in the respective figure legends.

## Results

### GSK3 Isozymes Are Essential for Accurate mTOR Signaling in Mature Neurons Cultured In Vitro

**Fig. 1:**
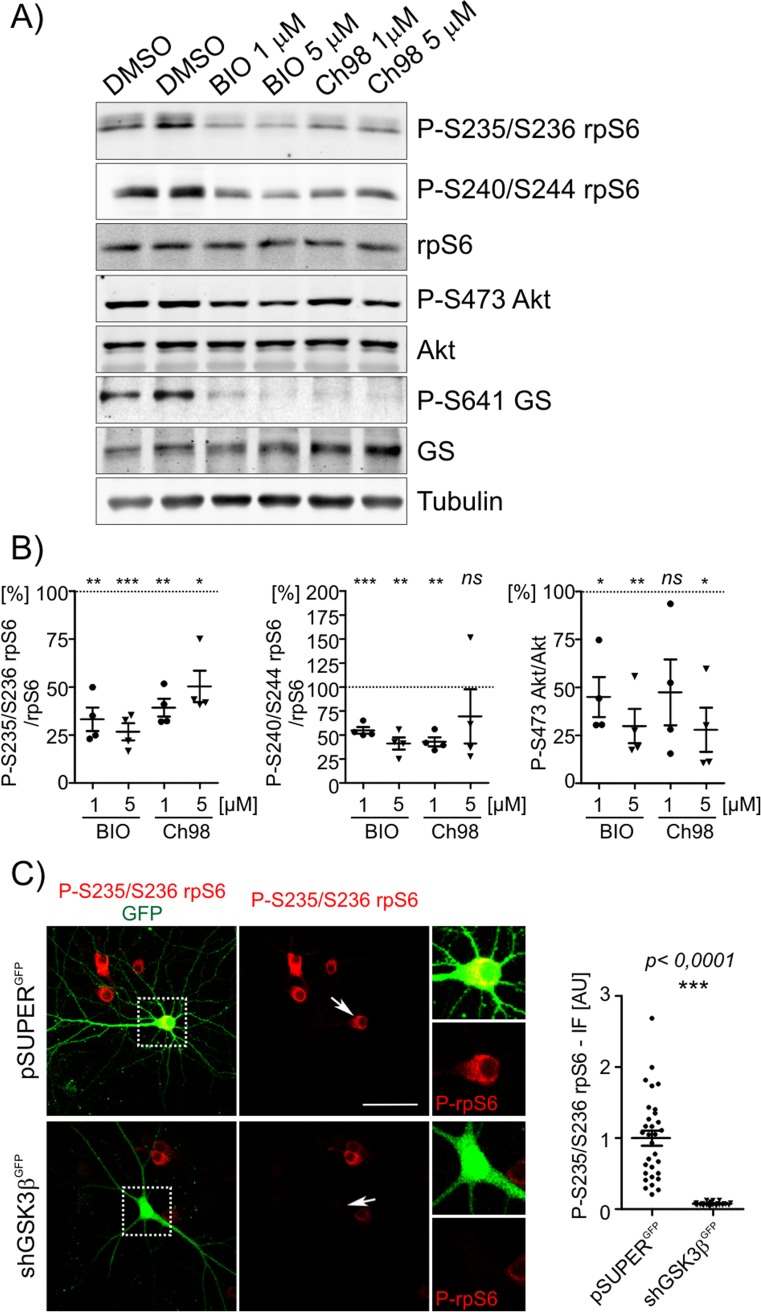
GSK3 activity is required for basal rpS6 and Akt phosphorylation in mature neurons cultured in vitro. **a** Fourteen-day in vitro neurons were treated for 2 h with the indicated GSK3 inhibitors. Western blot was used to determine the levels of P-S235/S236-rpS6, P-S240/S244-rpS6, rpS6, P-S473-Akt, Akt, P-S641-GS and GS. Tubulin is shown as a loading control. **b** WB analysis results. The signal was normalized to control levels (illustrated as a line). Error bars indicate SEM. ***p < 0.001, **p < 0.01, *p < 0.05 (one-sample *t* test). **c** Hippocampal neurons cultured in vitro were transfected on DIV14 for 4 days with either control pSUPER^GFP^ vector (*n* = 31) or pSUPER^GFP^ that encoded shRNAs against GSK3β (*n* = 25). Afterward, the cells were stained with an antibody against endogenous P-S235/S236-rpS6, and the average intensity of fluorescence was quantified (in arbitrary units [AU]). Error bars indicate SEM. ***p < 0.001 (unpaired *t* test). Scale bar = 50 μm

GSK3 isozymes were previously shown to inhibit or activate mTORC1, depending on the cellular context [5–7, 26]. The negative impact of GSK3β on mTORC2 was also reported in non-neuronal cells [4]. To clarify the impact of GSK3α and GSK3β on mTOR signaling pathways in mature neurons, mouse embryonic cortical neurons were cultured and challenged in vitro. On DIV14, cultures were treated with the structurally unrelated GSK3 isozyme inhibitors, Ch98 and BIO (both 1 and 5 μM), for 2 h. The degree of mTORC1 and mTORC2 activity was assessed by Western blot (WB) using fluorescently labeled secondary antibodies and the LI-COR Odyssey Imaging System. To test activity of mTORC1, the phosphorylation level of two of its downstream effectors: phosphorylated ribosomal protein (rp) S6 and eukaryotic translation initiation factor 4E-binding protein 1 (4E-BP1) (4E-BP1 [P-4E-BP1 [T37/T46]) was checked. In case of rpS6 phosphorylation two different antibodies, recognizing phosphorylated Ser235/Ser236 (P-S235/S236-rpS6) and Ser240/244 (P-S240/S244-rpS6), respectively, were used. Phosphorylated Akt (P-S473 Akt) served as a readout for mTORC2 activity. To determine drug efficacy, we measured the level of phosphorylated glycogen synthase (P-GS), the most well-defined GSK3 substrate. Both inhibitors substantially reduced P-S235/S236-rpS6, P-Ser240/244-rpS6 and P-Akt levels (Fig. 1a, b). In case of P-4E-BP1 the statistically significant decrease was observed only in case of Ch98 treatment (Online Resource 1, Fig. S1A). Yet, due to technical reasons WB analysis of P-4E-BP1 level in neuronal extracts turned out the least reliable (Online Resource 1, Fig. S1) due to relatively weak signal. To further confirm the requirement of GSK3β for sustaining basal P-S235/S236-rpS6 levels, DIV14 hippocampal mouse neurons were transfected with either empty pSuper^GFP^ (control) or a previously validated pool of pSuper^GFP^ plasmids that encode GSK3β short-hairpin RNAs (shRNAs) [19]. Three days post-transfection, the cells were fixed and immunofluorescently stained for P-rpS6 to reveal that GSK3β knockdown significantly decreased P-rpS6 levels (Fig. 1c).

**Fig. 2:**
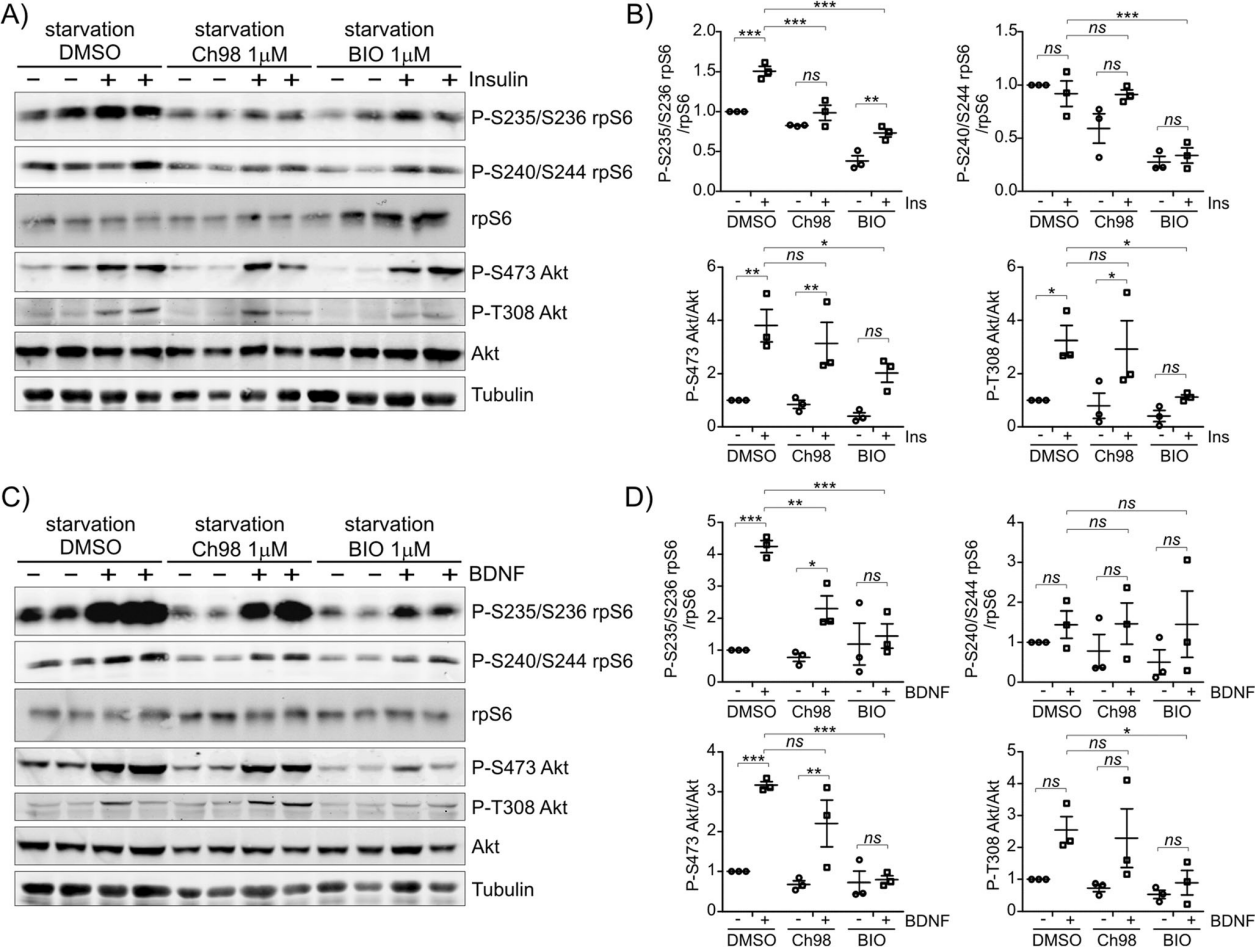
Effects of GSK3 inhibition on the induction of rpS6 and Akt phosphorylation in mature neurons cultured in vitro. Fourteen-day in vitro neurons were cultured overnight under conditions of trophic factor starvation (0.2% B27) and then treated for 2 h with the indicated GSK3 inhibitors prior to 20-min stimulation with (**a**, **b**) insulin (400 nM) or (**c**, **d**) BDNF (100 ng/ml). Western blot was used to determine the levels of P-S235/S236-rpS6, P-S240/S244-rpS6, rpS6, P-S473-Akt, P-T308-Akt and Akt. Tubulin is shown as a loading control. (**b**, **d**) WB analysis results. The signal was normalized to control levels (illustrated as a line). Error bars indicate SEM. ***p < 0.001, **p < 0.01, *p < 0.05 (Two*-*Way ANOVA with Bonferroni correction)

In neurons, mTOR complexes activity is potentiated by several stimuli, including BDNF [27, 28] and insulin [24, 29]. Thus, we tested whether GSK3α and GSK3β are required for mTORC1 and mTORC2 effector activation in response to such stimulation. DIV14 cortical neurons were cultured overnight in medium with decreased concentrations of the B27 supplement, to synchronize mTOR complexes activity at low level. The cultures were then treated with BDNF or insulin for 20 min combined with GSK3 inhibition. In case of these starvation-acute restimulation experiments, results of WB analysis were less straightforward than under basal conditions. For example, starvation resulted in less pronounced and more diverse effects of GSK3 inhibition on all analyzed phosphosites (Fig. 2 and Fig. S1 [Online Resource 1]). Both acute insulin and BDNF treatment potentiated phosphorylation of rpS6 at Ser235/Ser236 and Akt at S473 and T308 (indicator of Akt activation by PI3K-PDK1 pathway). On the other hand, P-S240/S244-rpS6 level did not increase in response to insulin and was only slightly, although statistically insignificantly, increased in response to BDNF. GSK3 inhibition by CH98, with an exception of upregulation of P-S235/S236-rpS6 induced by insulin, did not prevent ability of neuronal stimulation to increase investigated residues phosphorylation, although some inhibitory effect could be observed (Fig. 2a–d). On the other hand, BIO was more potent and in most cases, degree of the inhibition reached statistical significance (Fig. 2a–d). These results indicated that GSK3α and GSK3β were needed to sustain the physiological activity of mTORC1 and mTORC2 under basal conditions. However, under starvation-acute restimulation experimental paradigms the positive effects of GSK3 on mTOR signaling were less pronounced, if any.

### GSK3β Regulates mTOR Activity in the Brain In Vivo

**Fig. 3:**
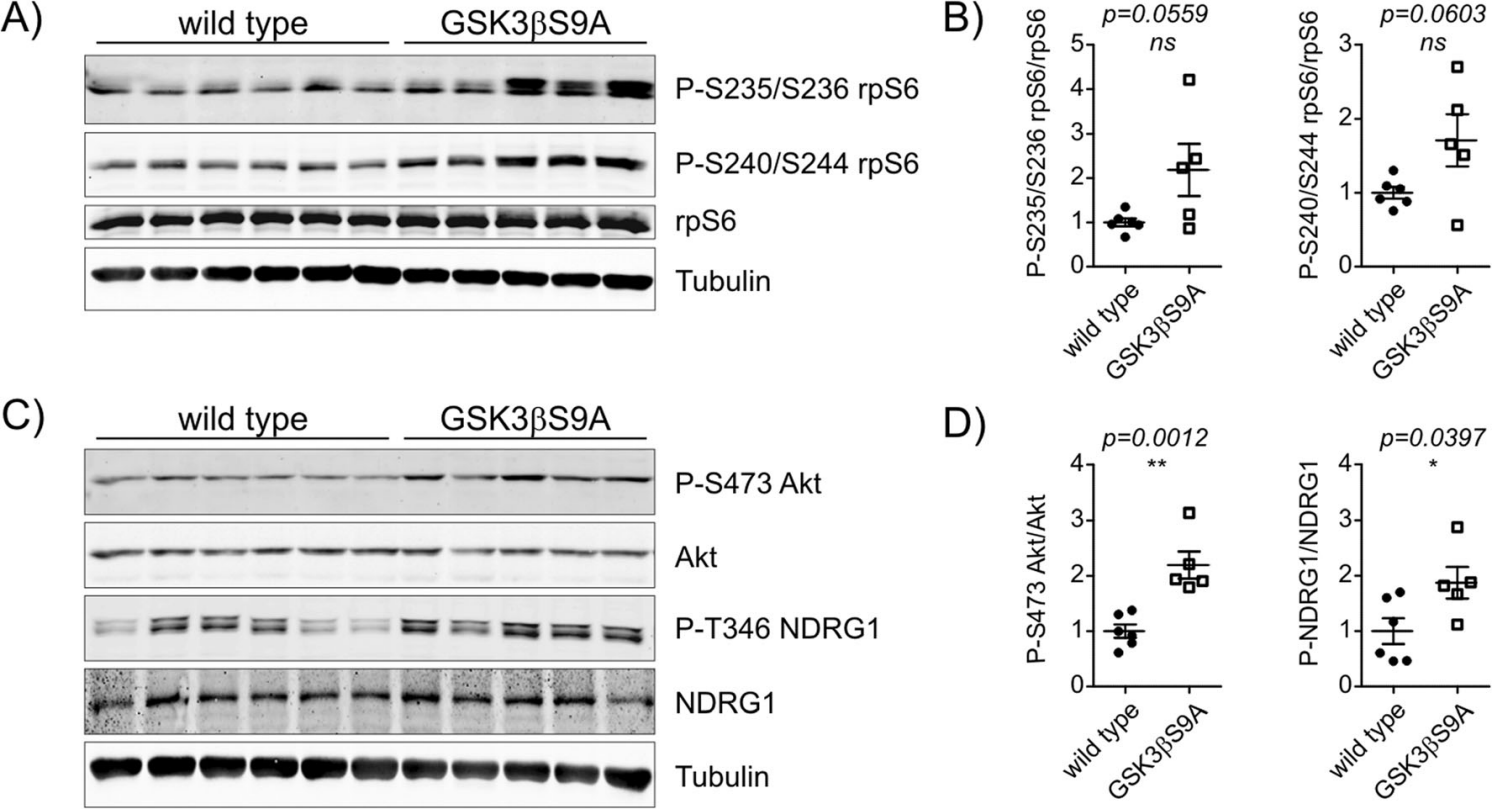
GSK3β activity potentiates the phosphorylation of mTORC2 effectors in vivo under basal conditions. **a** The hippocampi from wildtype (*n* = 6) and GSK3β S9A (*n* = 5) mice were isolated and lysed. Western blot was used to determine the levels of P-S235/S236-rpS6, P-S240/S244-rpS6 and rpS6. Tubulin is shown as a loading control. **b** WB analysis results. **c** Western blot was used to determine the levels of P-S473-Akt, Akt, P-Thr346-NDRG1 and NDRG1. Tubulin is shown as a loading control. **d** WB analysis results. Error bars indicate SEM. **p < 0.01, *p < 0.05 (unpaired *t* test)

To confirm that the observed positive impact of GSK3β on mTORC1 and mTORC2 effectors was not restricted to cultured neurons, we examined the way in which this kinase affects their phosphorylation in the brain in vivo. We used transgenic mice with postnatal neuron-specific expression of constitutively active GSK3β (GSK3β S9A) and mice with postnatal neuron-specific knockout of GSK3β (GSK3β[n−/−]) to mimic in vivo conditions of increased and decreased activity of GSK3β, respectively. Under basal conditions, levels of P-S235/S236-rpS6 and P-S240/S244-rpS6 in hippocampal lysates from 4-month-old GSK3β S9A mice were not significantly higher than in age-matched wildtype mice (Fig. 3a, b). We also evaluated the phosphorylation status of P-4E-BP1 and we observed no differences but obtained signal was of poor quality (Online Resource 1, Fig. S1). mTORC2 signaling was upregulated in the brain of GSK3 S9A mice as reflected by the increased levels of its effectors P-Akt and phosphorylated N-Myc Downstream Regulated 1 (P-NDRG1 [Thr346]; Fig. 3c, d).

**Fig. 4:**
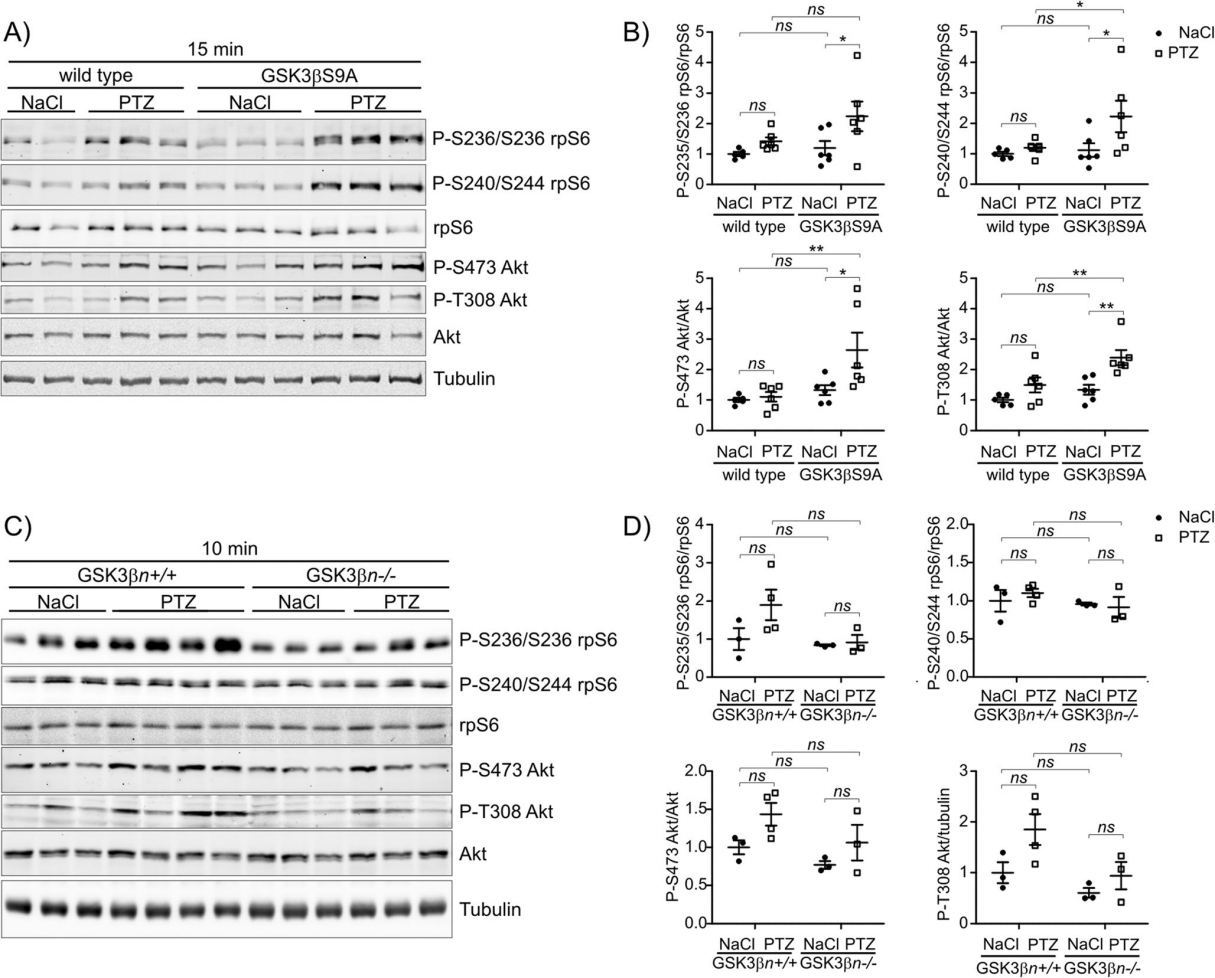
GSK3β activity potentiates the phosphorylation of mTORC1 and mTORC2 effectors in vivo upon increases in neuronal excitation. **a** Wildtype and GSK3β S9A mice were given NaCl (wildtype, *n* = 5; GSK3β S9A, *n* = 6) or PTZ (50 mg/kg; wildtype, *n* = 6; GSK3β S9A, *n* = 6). After 15 min, the hippocampi were isolated and lysed. Western blot was used to determine the levels of P-S235/S236-rpS6, P-S240/S244-rpS6, rpS6, P-S473-Akt, P-T308-Akt and Akt. Tubulin is shown as a loading control. **b** WB analysis results. **c** GSK3β(n+/+) and GSK3β(n−/−) mice were administered NaCl (GSK3β[n+/+], *n* = 3; GSK3β[n−/−], *n* = 3) or PTZ (30 mg/kg; GSK3β[n+/+], *n* = 4; GSK3β[n−/−], *n* = 3). After 10 min, the hippocampi were isolated and lysed. Western blot was used to determine the levels of P-S235/S236-rpS6, P-S240/S244-rpS6, rpS6, P-S473-Akt, P-T308-Akt and Akt. Tubulin is shown as a loading control. **d** WB analysis results. Error bars indicate SEM. **p < 0.01, *p < 0.05 (two*-*way ANOVA with Bonferroni correction)

mTOR is activated in vivo by proconvulsive neuronal stimulation [30–33]. Therefore, young, 4-month-old, wildtype and GSK3β S9A mice were administered PTZ (50 mg/kg), an inhibitor of GABAergic transmission that induces generalized seizures and acutely activates mTORC1 [33]. Control mice of both genotypes were treated with 0.9% NaCl. Fifteen minutes after seizure onset, the mice were sacrificed, their hippocampi isolated and the P-rpS6 and P-Akt levels analyzed by WB on protein lysates. Analysis revealed that in GSK3β S9A mice, PTZ caused much larger increase of P-S235/S236-rpS6, P-S240/S244-rpS6 and P-S473-Akt than in wild type mice (Fig. 4a, b). Again analysis of P-4E-BP1 turned out inconclusive (Online Resource 1, Fig. S1). Altogether this suggests that increased activity of GSK3β leads to more robust activity of mTORC1 and mTORC2 in response to robust excitatory neuronal stimulation.

Next, we evaluated whether GSK3β is needed for the phosphorylation of rpS6 and Akt in vivo. Indeed, we observed slightly lower P-rpS6 and P-Akt levels in the hippocampus in GSK3β(n−/−) mice compared with their GSK3β^fl/fl^ littermates, but this difference was not statistically significant (Fig. 4c, d). We also treated GSKβ(n−/−) and GSK3β^fl/fl^ mice similarly to the GSK3β S9A mice with PTZ. However, PTZ at a dose of 50 mg/kg caused death of all GSK3β(n−/−) mice immediately after the first seizure episode (4/4 PTZ-injected mice), whereas the control GSK3β^fl/fl^ littermates developed seizures and survived similarly to wildtype mice (6/6 PTZ-injected mice). Therefore, we injected lower doses of PTZ (30 mg/kg) and shortened the experiment to 10 min postinjection to minimize mortality of the GSK3β-knockout mice. Even with this attenuated treatment regimen, only 50% of the GSK3β(n−/−) mice survived (3/6 mice) until 10 min p.i., at which time the surviving animals were sacrificed. Similarly to the previous experiments, control mice of both genotypes received saline injections. The analysis of the selected phospho-protein levels in hippocampal lysates from GSK3β^fl/fl^ and GSK3β(n−/−) mice did not reveled significant differences between genotypes in response to PTZ. However, some tendency for lower activation of P-S235/S236-rpS6, P-S473-Akt and P-T308-Akt was observed in knockout mice (Fig. 4c, d). Altogether, these data suggest that increased GSK3β activity exerts a positive effect on the mTORC1/S6K1 and mTORC2 pathways in vivo and can potentiate mTORCs response to PTZ. On the other hand, GSK3β is not critically needed for mTORC1/S6K1 and mTORC2 activity in vivo. Alternatively GSK3β is substituted by GSK3α.

### Neuronal Expression of GSK3β S9A Increases the Activation of Prosurvival Signaling Pathways and Attenuates Kainic Acid-Induced Apoptosis

**Fig. 5:**
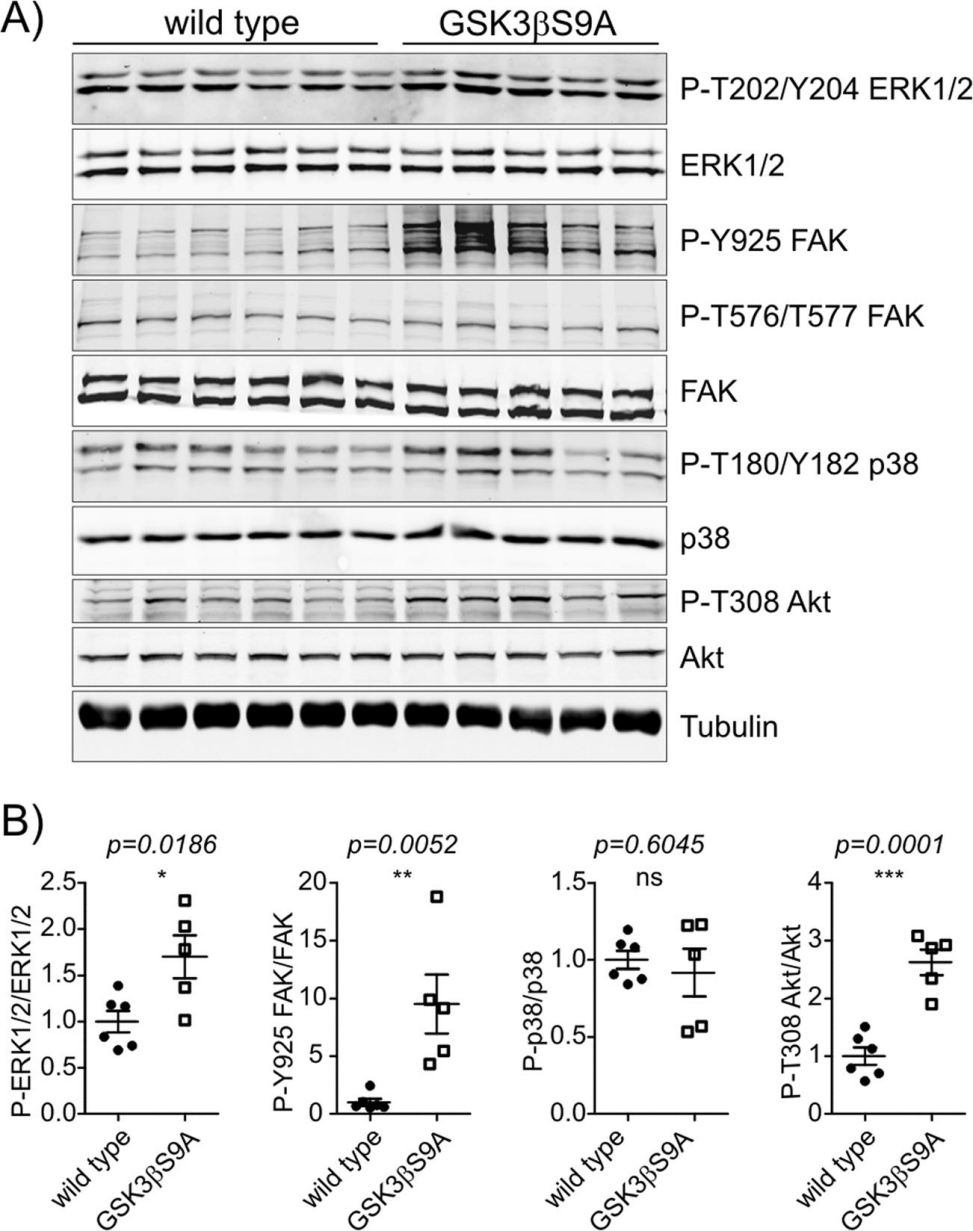
GSK3β increases prosurvival signaling in the brain. The hippocampi from wildtype (*n* = 6) and GSK3β S9A (*n* = 5) mice were isolated and lysed. **a** Western blot was used to determine the levels of P-ERK 1/2 (T202/Y204), ERK 1/2, P-FAK (Y925), P-FAK (T576/T577), FAK, P-p38 (T180/Y182), p-38, P-Akt (T308) and Akt. Tubulin is shown as a loading control. **b** WB analysis results. Error bars indicate SEM. ***p < 0.001, **p < 0.01, *p < 0.05 (unpaired *t* test)

The mTORC2 effector Akt is widely known for its involvement in prosurvival signaling. We analyzed additional signaling pathways that are related to cell survival in GSK3 S9A mice. GSK3β S9A mice had elevated hippocampal levels of phosphorylated extracellular signal-regulated kinase 1/2 (P-ERK1/2 [Thr202/Tyr204]), phosphorylated focal adhesion kinase (P-FAK [Tyr925]), but not phosphorylated p38 (P-p38 [Thr180/Tyr182]; Fig. 5a, b), suggesting that higher GSK3β activity can be neuroprotective [34–37].

**Fig. 6:**
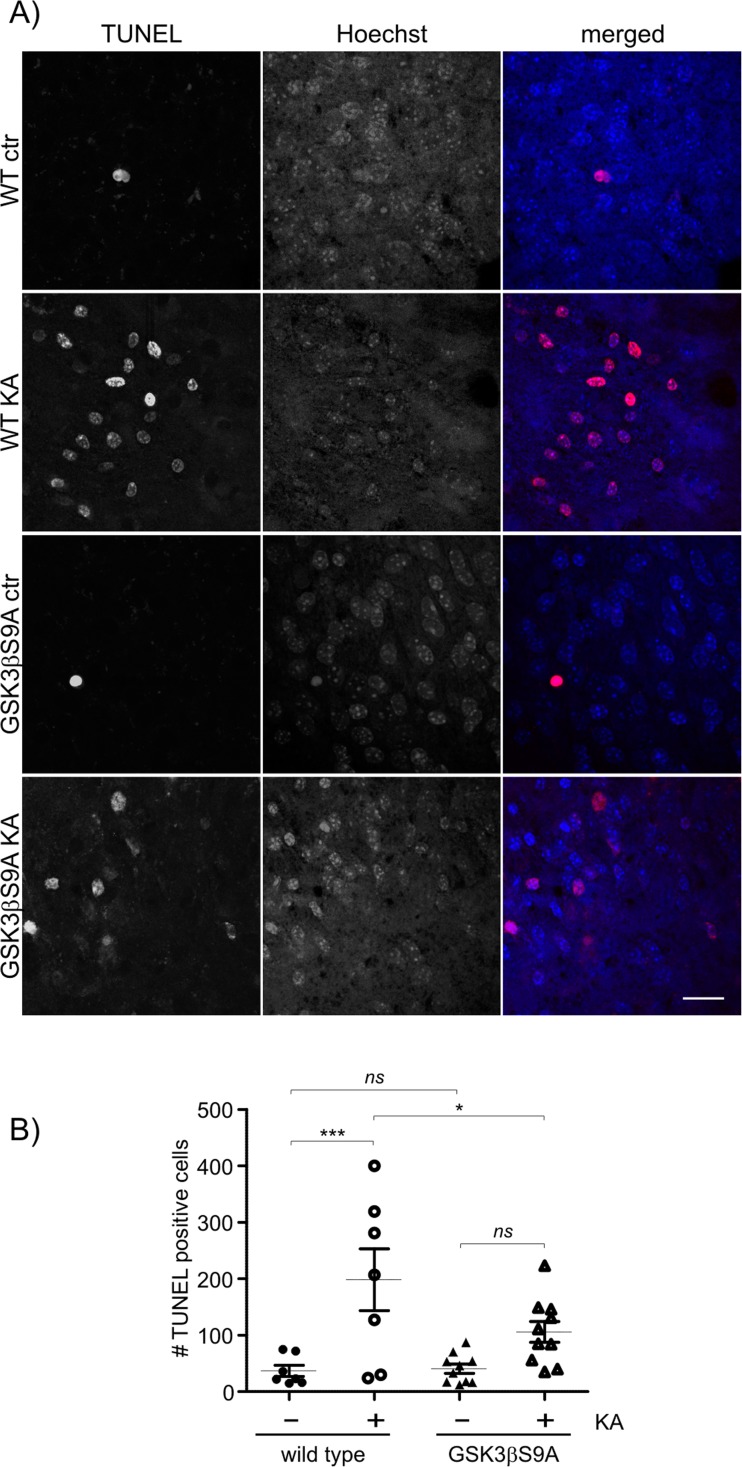
GSK3β S9A limits KA-induced cell death in organotypic hippocampal cultures in vitro. **a** Slices from wildtype (*n* = 7) and GSK3β S9A (*n* = 10) mice were exposed to all types of treatment: vehicle and KA (10 μm, 24 h). TUNEL staining was then performed. TUNEL-positive nuclei were counted automatically. Hoechst 33258 staining was performed to reveal all cell nuclei that were present in the image. Scale bar = 20 μm. **b** Quantitative analysis results. Error bars indicate SEM. ***p < 0.001, *p < 0.05 (two-way ANOVA with Bonferroni correction). Scale bar = 25 μm

We evaluated whether constitutively active GSK3β limits KA-induced apoptosis. We used KA-treated hippocampal organotypic cultures in vitro, a convenient experimental system that reliably recapitulates in vivo neuropathology and provides high reproducibility in molecular analyses [25]. Organotypic hippocampal slices were obtained from newborn wildtype and GSK3β S9A mice and cultured for 10–14 days before being treated with KA for 24 h. The slices were stained by TUNEL to identify dying cells. KA substantially increased the number of TUNEL-positive cells in slices from wildtype mice compared with vehicle-treated slices, indicating increased cell death (Fig. 6). The effect of KA on cell death in slices from GSK3 S9A mice was less pronounced (Fig. 6). These results led us to conclude that GSK3β protected neurons from KA-induced cell death.

## Discussion

In the present study, we observed in vivo and in neurons cultured in vitro, that under certain conditions GSK3 positively regulated the phosphorylation of mTORC1 and mTORC2 effectors. For example, the decreased activity of GSK3α and GSK3β resulted in lower phosphorylation of rpS6 (S235/236 and S240/244) and Akt (S473) under basal conditions in vitro. In contrast an increase in neuronal GSK3β activity potentiated phosphorylation of downstream effectors of mTORC1 and mTORC2 pathways, both under basal conditions and in response to increased excitatory neurotransmission. Finally, we provided evidence that GSK3β upregulated additional prosurvival signaling pathways in vivo and reduced KA-induced cell death in organotypic hippocampal slices. The combined data-sets suggest that GSK3β may act as a positive regulator of mTOR and prosurvival signaling pathways in neurons.

GSK3α and GSK3β act as either negative or positive regulators of mTORC1 signaling, depending on the experimental conditions [5, 7, 26, 38]. In the present study, we report that GSK3α and GSK3β are needed for phosphorylation of the mTORCs effectors rpS6 and Akt in mature neurons. These results are consistent with previous studies that evaluated MCF-7 cells, mouse embryonic fibroblasts, and SH-SY5Y human neuroblastoma cells [6, 7]. However, our findings differ from those of Ma et al. [26], who reported increased S6K1 activation in hippocampal slices upon GSK3 inhibition with lithium ions or with kenpaullone. Notably, both lithium salts and kenpaullone are known to exert more pleiotropic effects than Ch98 and BIO used in our current studies. For example, lithium ions can inhibit GSK3 through multiple mechanisms that are not fully understood, including enhancing inhibitory GSK3 auto-phosphorylation or by protein kinase A, protein kinase C, and Akt [39]. Kenpaullone inhibits cyclin-dependent kinases (CDK1, CDK2, CDK5) and lymphocyte kinases, in addition to inhibiting GSK3 (http://www.selleckchem.com). Thus, the effects of GSK3 on mTOR signaling may depend on the initial state of cellular signaling networks. In this regard balance between activity of kinases positively and negatively regulating Tuberous Sclerosis Complex 2 (Tsc2) might be especially important. Tsc2 is a negative regulator of mTORC1 and Tsc2 activity has been shown in HEK293 cells to be potentiated by GSK3β [5]. Yet, this canonical way of GSK3β-driven inactivation of mTORC1 requires priming phosphorylation of Tsc2 by 5’AMP-activated protein kinase (AMPK), activity of which depends on energy status of the cell. What is more, increased activation of GSK3β in neutrophils and macrophages eventually leads to inhibition of AMPK [40]. Thus, it is fair to speculate that HEK293 cells and neurons differ in their steady state levels of AMPK activity and consequently in their susceptibility to GSK3β-Tsc2-driven inhibition of mTORC1.

Under certain conditions, GSK3 isozymes may upregulate mTORC1 signaling, and the mechanism may involve Akt as upstream regulator of mTORC1. Indeed, we found that P-Akt levels were higher in brain of GSK3β S9A mice treated with PTZ, in which P-rpS6 was also elevated. These findings are consistent with another study, in which GSK3 isozymes were reported to be needed for Akt phosphorylation in specified cell lines [8]. The different ways in which GSK3 positively regulates mTORC1 activity remain elusive and require further studies. For example, GSK3β may activate mTORC1 through direct Raptor phosphorylation, which was, however, described only in the context of mTORC1 regulation by amino acids [41]. Finally, we cannot exclude direct effects of GSK3 on 4E-BP1 and S6K1 phosphorylation. Indeed GSK3β can phosphorylate S6K1 at Ser371 [8], which is required for the subsequent phosphorylation of S6K1 Thr389 by mTORC1 and the activation of S6K1. GSK3β also directly phosphorylates 4E-BP1 at Thr37/Thr46 and activates this protein even when mTORC1 is inhibited [42]. Thus, it is possible that in neurons, GSK3 has synergistic effects with mTORC1 under certain conditions, rather than acting upstream of mTORC1.

In addition to positive effects of GSK3 on mTORCs we reported here that in brains of GSK3S9A mouse prosurvival signaling is upregulated. Consequently we demonstrated lower KA-induced cell death in hippocampal slices from GSK3S9A mouse. While this result does not contradict previous reports on GSK3 S9A mice, it is in sharp contrast with results obtained with use of TetO-GSK3βxCamKII-tTA-Cre mice in which, GSK3β cellular levels could be rapidly increased in mature forebrain neurons, resulting in neuronal cell death. Also several data from in vitro cultured neurons argue against anti-apoptotic function of GSK3β. But in vitro studies involving GSK3β inhibition or overexpression are acute in their nature and cannot be reliably compared with transgenic GSK3S9A overexpression in vivo. Also transient transfection typically results in higher cellular levels of protein of interest than transgenesis. Thus, the difference between phenotype induced by GSK3 S9A overexpression in vitro and in vivo could simply reflect differences in the level of GSK3β activity. But such difference could not explain discrepancy between in vivo models mentioned above. On the other hand, these models differ in timing of transgene expression, cell population expressing transgene and transgene itself (wild type GSK3β vs. GSK3β S9A). It is quite likely that these differences combination underlie opposite effects of GSK3β activity on neuronal survival in vivo reported by Lucas et al. [11] and us, suggesting that relatively early and mild overexpression of GSK3S9A induces cellular signaling adjustments that eventually protect them from excitotoxic injury. This can be achieved either by upregulation of prosurvival signaling pathways and/or by changes in neuronal excitation at the synaptic levels. In fact, in some cell types GSK3β can act as prosurvival factor although the exact mechanism is not well understood [43]. In non-neuronal cells, such prosurvival GSK3β actions, most likely, involve selective activation of anti-apoptotic gene transcription (e.g., *Bcl-2*) by nuclear factor κB (NFκB; [43]). Although protective role of NFκB in excitotoxic neuronal cell death is still debated, data from neurons lacking its critical subunit - p50, argue for it [44, 45]. Thus, one could speculate that in already developed Thy1-GSK3β S9A mice neurons, prolonged and moderate activation of GSK3 favors its prosurvial potential (e.g., activation of prosurvival signal transduction [via Akt] or gene expression [via NFκB]), which protects them against KA-triggered toxicity. On the other hand, GSK3 isozmes are known to regulate both excitatory and inhibitory synaptic transmission, thus increased threshold for neuronal excitation could prevent KA-induced excitoxicity [46–49]. Thus, further studies are warranted to understand relative resistance of GSK3S9A overexpressing neurons to excitotoxic stimulation by KA.

## Electronic Supplementary Material

Fig. S1 (PDF 169 kb)
