# Supplementary material for: GSK3β Controls mTOR and Prosurvival Signaling in Neurons
Source: Mol Neurobiol. 2017 Nov 15;55(7):6050–62. doi: 10.1007/s12035-017-0823-9 (PMC5994211; doi:10.1007/s12035-017-0823-9)
Supplement: Supplementary file 1 — (PDF 169 kb) [file 12035_2017_823_MOESM1_ESM.pdf]

Urbanska et al. - Online Resource 1. Supplementary Figures

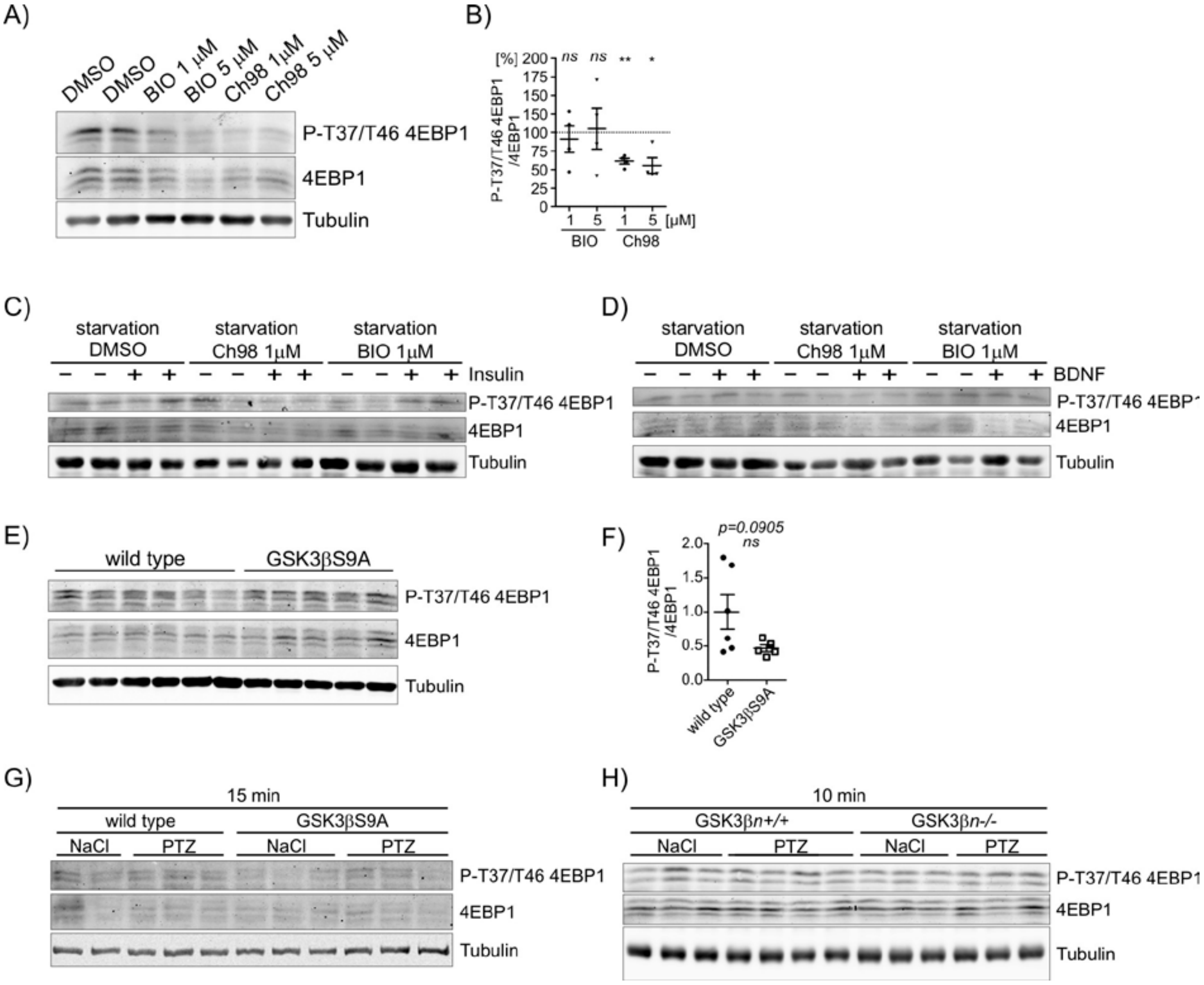

Figure S1. WB analysis of P-T37/T46 4E-BP1 in experiments presented in **(A, B)** Fig. 1, **(C, D)** Fig. 2, **(E, F)** Fig. 3, **(G)** Fig. 4 and **(H)** Fig. 5.
